# Individual cognitive empowerment and in-role performance: a matched-pair study

**DOI:** 10.3389/fpsyg.2024.1402029

**Published:** 2024-09-16

**Authors:** Jeniboy Kimpah, Sebastiaan Rothmann, Hazril Izwar Ibrahim, Amar Hisham Jaaffar, Cinthia Annisa Vinahapsari, Walton Wider, Lester Naces Udang

**Affiliations:** ^1^Faculty of Business and Communications, INTI International University, Nilai, Malaysia; ^2^Optentia Research Unit, North-West University, Vanderbijlpark, South Africa; ^3^School of Management, Universiti Sains Malaysia, Pulau Pinang, Malaysia; ^4^Institute of Energy Policy and Research (IEPRe), Universiti Tenaga Nasional, Kajang, Malaysia; ^5^Faculty of Liberal Arts, Shinawatra University, Pathum Thani, Thailand; ^6^College of Education, University of the Philipphines, Diliman, Philippines

**Keywords:** environmental empowerment, in-role performance, E&E manufacturing sector, economic productivity, cognitive empowerment

## Abstract

The study examines the model fit of individual cognitive empowerment, which includes psychological and environmental factors, and investigates the direct effect of environmental empowerment specifically work methods, work criteria, and work schedules on in-role performance in Malaysia’s Electrical and Electronic (E&E) manufacturing firms. Data were obtained from 173 engineers and 173 managers, matched in pairs, who have worked together for at least 1 year in 73 E&E manufacturing firms. The study found that the model fit of psychological and environmental factors is stronger for individual cognitive empowerment. Work methods show a positive direct effect on in-role performance. However, work schedules and work criteria do not appear to have a direct effect on in-role performance. This study highlights the importance of considering work methods in the engagement between engineer and manager pairs in the E&E manufacturing sector to enhance in-role performance.

## 1 Introduction

The study faced criticism both theoretically and methodologically, particularly in its examination of environmental factors shaping individual cognitive empowerment. This study critiques the theoretical aspects in reexamining individual cognitive empowerment with a focus on two components: psychological and environmental factors. Psychological factors pertain to psychological empowerment, while environmental factors pertain to environmental empowerment. Past research on individual cognitive empowerment through psychological empowerment is well established (Raub and Robert, 2010; Seibert et al., 2011; Alhozi et al., 2021; Zhang et al., 2022).

However, studies on environmental empowerment are lacking. This gap was highlighted by Meyerson and Kline (2008), who identified a need for more research on individual cognitive empowerment between psychological and environmental empowerment. Secondly, this study critiques the methodological aspects. Most past studies were conducted using single sources, but this study employed paired sources to assess the effect of individual cognitive empowerment and in-role performance. We utilized a match-pair design, pairing superiors with subordinates (Nekooie, 2021; Quigley et al., 2022). This study involves a match-pair design, particularly a dyadic relationship between engineers and managers, who are pairing of a one-to-one matched pair.

The presence of psychological and environmental factors is critical in addressing gaps in strengthening a model of individual cognitive empowerment and in promoting employee empowerment sustainability. This study has found that psychological factors relate to psychological empowerment (Spreitzer, 1995) and environmental factors to environmental empowerment (Breaugh, 1985; Meyerson, 2007; Meyerson and Kline, 2008). Psychological empowerment refers to the feeling of control and influence over one’s work (Spreitzer, 1995). While environmental empowerment refers to the availability of resources and support in the work environment, especially regarding work methods, work schedules, and work criteria (Breaugh, 1985; Meyerson, 2007; Meyerson and Kline, 2008). Work methods, work schedules, and work criteria can be a motivation for autonomy and self-determination in work tasks and activities or, conversely, limit an individual’s ability to control.

Studies by Meyerson (2007) and Meyerson and Kline (2008) revealed that individual cognitive empowerment had a positive effect on the construct of psychological empowerment and environmental empowerment among part-time employees in Canada. A more in-depth analysis of the re-examination of the model fit of psychological empowerment and environmental empowerment is crucial for establishing the conceptual clarity of individual cognitive empowerment in different research contexts and approaches. The study was conducted within the context of the engineering sector.

The research gap exists in examining the significance of individual cognitive empowerment due to the lack of research conducted on the model fit of psychological empowerment and environmental empowerment on individual cognitive empowerment in the engineering sector (Fragkos et al., 2020). Past studies revealed the role of psychological empowerment in agile practices and performance, which could be relevant to examine individual cognitive empowerment in the engineering sector (Malik et al., 2021). However, there are limited studies that explore the relationship between environmental empowerment and performance in the engineering sector. Therefore, examining the relationship between environmental empowerment and performance, particularly in-role performance, is crucial for examining individual cognitive empowerment. This study aims to address the gap through a deeper analysis of the relationship between environmental empowerment and in-role performance using a one-to-one matched pair design within the engineering sector.

The theoretical justification for examining the model fit of psychological and environmental factors on individual cognitive empowerment lies in the need to examine the comprehensive influence of both intrinsic (psychological) and extrinsic (environmental) factors on cognitive individual empowerment. This dual perspective advances the current belief in integrating environmental factors, which have been underexplored, into the well-established framework of psychological empowerment. It provides a more holistic view of how different empowerment dimensions interact and contribute to in-role performance, offering practical in enhancing workplace effectiveness.

This research design made the study more relevant to the manufacturing industry, due to the challenging general issues of employee turnover (Ng et al., 2019). The study focuses on Malaysia’s Electrical and Electronic (E&E) sector is expected to generate RM495 billion in export earnings by 2025, as Malaysia continues to attract high-quality investments and is expected to uphold its growth trajectory (Bernama, 2022). In addition to its economic contribution, the E&E sector is a major employer in Malaysia, providing employment to over 590,000 employees (Van Cutsem et al., 2017; Huong, 2023).

Given the importance of the E&E sector, it is important to understand the factors that contribute to employee sustainability. A major focus of the study was, therefore, engineers’ work methods, work schedules, and work criteria and in-role performance due to their critical role in driving innovation, improving efficiency, and ensuring quality in the sector. Furthermore, the Engineering Accreditation Council (2021) emphasizes the importance of developing engineers with the necessary skills and abilities to meet the requirements of the sector.

This study aims to examine the impact of individual cognitive empowerment on in-role performance among engineers in Malaysia’s Electrical and Electronics (E&E) manufacturing sector. It evaluates the model fit of individual cognitive empowerment, focusing on psychological and environmental factors, and investigates the direct effect of environmental empowerment factors, such as work methods, work criteria, and work schedules on in-role performance. The study utilizes a matched-pair design involving engineers and managers from E&E manufacturing firms in Malaysia, filling a research gap in examining both psychological and environmental empowerment factors, thereby contributing to organizational psychology and offering valuable for the E&E manufacturing sector.

### 1.1 Individual cognitive empowerment

Empowerment is a concept that has been extensively studied and can be categorized into two distinct categories: individual cognitive empowerment and organizational structural empowerment. According to Amundsen and Martinsen (2015), individual cognitive empowerment is based on the work conditions of individuals with regard to intrinsic motivational (psychological) and relational (environmental) factors. Furthermore, organizational structural empowerment is concerned with formal power structures within an organization and their influence on employee perceptions of the workplace. The study, with its specific focus on individual cognitive empowerment, aims to make two significant contributions to the existing body of knowledge:

- *Re-examination of model fit*: To provide a more comprehensive understanding of individual cognitive empowerment, the study has re-examined the model fit of psychological and environmental factors on individual cognitive empowerment. Psychological factors refer to psychological empowerment, while environmental factors, encompassing work methods, work criteria, and work schedules, refer to environmental empowerment.
- *Impact on in-role performance*: Building upon the established positive association between environmental empowerment and individual cognitive empowerment (Thomas and Velthouse, 1990; Spreitzer, 1995), the study has delved deeper into this relationship in investigating the direct impact of environmental empowerment on in-role performance. In employing matched pairs, the study has sought to uncover the direct positive relationship between work methods, work criteria, and work schedules, and in-role performance.

### 1.2 Re-examining the two-factor model fit of individual cognitive empowerment: psychological and environmental factors

An ideal construct for psychological empowerment has rarely been present in the context of human resource practitioners in E&E manufacturing firms. Nonetheless, the study has supported psychological empowerment with productivity, profitability, and workplace effectiveness. Additional empowerment factors, namely psychological and environmental, must be considered to achieve better job outcomes in terms of intrinsic motivation and relationships. The cited studies have provided evidence that contextual antecedent constructs, positive self-evaluation traits, structural and psychological empowerment, and psychological empowerment are positively related to job outcomes, particularly productivity, profitability, workplace effectiveness, work motivation, occupational mental health, and job performance (Van Cutsem et al., 2017).

Studies have emphasized the crucial influence of psychological empowerment on job outcomes. The most consistently used measurement of psychological empowerment consists of four factors derived from the concept of intrinsic motivation: perceived self-determination, perceived meaning, perceived competence, and perceived impact (Seibert et al., 2011). Profit and non-profit organizations have tested these factors to determine job outcomes. Seibert et al. (2011) provide meta-analytical support for an integrated model specifying the antecedents and consequences of psychological and team empowerment. Meira and Hancer (2021) developed a conceptual model for the hospitality industry based on the employee-organization relationship using the social exchange theory. The study considered perceived organizational support to be a psychological empowerment antecedent, while work engagement and service- oriented organizational citizenship behavior were considered its outcome.

Spreitzer (1995) developed the most consistently used measurement of psychological empowerment. It comprises four factors, mentioned above, derived from the concept of intrinsic motivation: perceived self-determination, perceived meaning, perceived competence, and perceived impact. Perceived self-determination refers to an individual’s sense of autonomy and control over work. Perceived meaning refers to an individual’s belief that work is meaningful and has a purpose. Perceived competence relates to an individual’s belief in his/her ability to perform a task successfully. Perceived impact relates to an individual’s belief that his/her work has an impact and makes a difference.

These findings are supported by Meyerson (2007) and Meyerson and Kline (2008), who conducted a study into individual cognitive empowerment, specifically psychological empowerment measurements, and found that Spreitzer’s psychological empowerment scale from 1995 had a scarcity of items related to autonomous behavior, management practice, and relational factors. This limitation might affect employee performance sustainability through the ability of an employee to maintain productivity. To address this limitation, Meyerson (2007) and Meyerson and Kline (2008) re-examined psychological empowerment, with a focus on the self-determination dimension on Spreitzer’s psychological empowerment scale. Although Spreitzer et al. (1997) suggested further study into the encompassing measures of the self-determination dimension, Meyerson (2007) and Meyerson and Kline (2008) found that the self-determination scale had a limited effect in relation to autonomous behavior, management practice, and relational factors.

In addressing the research gap, Meyerson (2007) and Meyerson and Kline (2008) determined that these items were linked to Breaugh’s autonomy behavior scale (1985). This led Meyerson (2007) and Meyerson and Kline (2008) to redefine Breaugh’s autonomy behavior scale (1985). It had a suitable fit with Spreitzer’s self-determination scale. Thus, the study decided to exclude Spreitzer’s self-determination scale due to its inherent collinearity link with autonomous behavior. The proof was provided by Meyerson (2007) and further supported by Meyerson and Kline (2008), who found that Breaugh’s autonomy behavior scale, autonomous behavior, management practice, and relational factors. Furthermore, Lee and Koh (2001) supported the belief that autonomous behavior was not separate from the empowerment measurement.

Studies by Meyerson (2007) and Meyerson and Kline (2008) identified several biases related to autonomous behavior, management practice, and relational factors in Spreitzer’s psychological empowerment scale. Meyerson (2007) and Meyerson and Kline (2008) described the results of re-examining Spreitzer’s psychological empowerment scale from the perspectives of perceived meaning, perceived competence, and perceived impact. However, the perceived self-determination scale was replaced the autonomous behavior, management practice, and relational factors scale developed by Breaugh (1985), which was later referred to as environmental empowerment by Meyerson (2007) and Meyerson and Kline (2008). It can be seen that Meyerson (2007) and Meyerson and Kline (2008) conducted a study to re-examine individual cognitive empowerment, the psychological empowerment scale (without the self-determination scale), and environmental empowerment.

According to Meyerson (2007) and Meyerson and Kline (2008), the two-factor model of individual cognitive empowerment (a combination of psychological empowerment scales [without self-determination scales] and environmental empowerment scales) was a better fit model and was more comprehensive in helping to improve the sustainability of employee performance. The results of these studies led to the following hypothesis:

*Hypothesis 1*: Psychological empowerment and environmental empowerment (the two-factor model) have a better fit model in measuring individual cognitive empowerment.

### 1.3 Relationship between environmental empowerment and in-role performance

The study has provided a novel perspective on the construct of individual cognitive empowerment within the context of human resource management, particularly focusing on the relationship between environmental empowerment and in-role performance in the workplace. Meyerson (2007) and Meyerson and Kline (2008) have defined environmental empowerment as the perception of situations through work environments or work conditions that enable individuals to make decisions about how work is conducted. Key factors contributing to environmental empowerment include information sharing, clear structures, commitment, flexible schedules, procedures, and independence in deciding work criteria.

To measure environmental empowerment, employees need to have control over work environment factors, including work methods, work schedules, and work criteria used in completing tasks. Environmental empowerment has been found to enhance employees’ sense of psychological comfort and control over work, leading to improve in-role performance. These constructs can be applied to create a more empowering work environment that benefits employees’ in-role performance. In-role performance pertains to an employee’s ability to fulfil the duties and responsibilities outlined in his/her job description, such as meeting deadlines, following procedures, achieving productivity, and meeting quality standards. Therefore, the study has investigated the relationship between work methods, work schedules, and work criteria, and in-role performance.

#### 1.3.1 Relationship between work methods and in-role performance

The relationship between work methods and employee in-role performance consistently demonstrates a positive correlation between the two. Nugroho’s (2021) empirical study revealed that transitioning from office-based work method to remote work during the COVID-19 pandemic positively impacted employee performance, highlighting the adaptability of work methods in the new normal era. Similarly, Trinova et al. (2021) indirectly suggested the role of work methods in shaping lecturer performance in examining the influence of diverse teaching approaches and the use of varied media. Further emphasizing the importance of adapting work methods to individual needs and capabilities, Adler and Borys (1996) advocated for a collaborative approach to time-and-motion analysis, integrating prescribed methods with employee improvisations to optimize productivity and performance. These findings collectively emphasize the significance of autonomy in determining suitable work methods for enhancing employee performance.

*Hypothesis 2*: Work methods have a positive significant effect on in-role performance.

#### 1.3.2 Relationship between work schedules and in-role performance

Several studies have investigated the relationship between work schedules and employee performance, with findings consistently suggesting a positive association between these two variables (Kandie and Chepkilot, 2022; Misnan et al., 2022). Effective work scheduling and prioritization have been found to enhance employee performance and mitigate the negative impacts of work schedule disorders (Kandie and Chepkilot, 2022). Safety cultures play a crucial role in moderating the effects of work schedules on performance, with a strong safety culture significantly reducing the detrimental effects of work schedule disorders (Al-Mekhlafi et al., 2022). Additionally, flexible work arrangements, such as flexible schedules, have shown a positive influence on employee performance (Misnan et al., 2022). These findings highlight the importance of considering work schedules in efforts to optimize employee performance.

*Hypothesis 3*: Work schedules have a positive significant effect on in-role performance.

#### 1.3.3 Relationship between work criteria and in-role performance

Dong et al. (2022) emphasize the importance of minimizing work criteria in production planning due to its critical impact on customer satisfaction and the need for managers to avoid financial losses. The literature discusses the complexities of integrating late work as an objective function and proposes various algorithms to address optimization problems across different industries. Lau and Aguirre Reid (2022) investigated the direct relationship between work method, work scheduling, work criteria, and employees’ performance in the context of organizational uncertainty. The findings support a positive association between employee work method, work scheduling, work criteria and performance, with work criteria identified as the strongest predictor.

*Hypothesis 4*: Work criteria have a positive significant effect on in-role performance.

## 2 Research design

### 2.1 Matched pair

Kenny and Winquist (2001) proposed a standard matched-pair design, pairing superiors with subordinates. This design actively involves a dyadic relationship between managers and engineers, who are members of a one-to-one matched pair. The study has specifically focused on the dyadic relationship between managers and engineers, considering them as matched pairs (Butt et al., 2023). This design enhances internal validity in controling for inter-individual differences, ensuring a more accurate assessment of individual cognitive empowerment on in-role performance without confounding effects from extraneous variables. This approach ensures that the observed effects are not confounded extraneous variables, thereby strengthening the study’s internal validity.

### 2.2 Population

The population of the study was drawn from all the employees in the Malaysian E&E manufacturing firms based on the products produced as categorized by the Federation of Malaysian Manufacturers (FMM). These firms are classified into five product groups: (1) office, accounting, and computing machinery; (2) domestic equipment; (3) radio, television, and communication equipment; (4) electrical machinery and apparatus; and (5) medical, precision, and optical instruments, including watches and clocks.

### 2.3 Unit of analysis

The unit of analysis for the study has been individuals working in E&E manufacturing firms, specifically managers and engineers. To be included in the study, each manager and engineer has had to work for the current company for at least 1 year, and each engineer has had to have a direct subordinate reporting to the manager, who is considered the coordinator or supervisor based on work outcome relationships.

Due to employing the research design of a matched pair, managers have been responsible for assessing the in-role performance of engineers, while engineers have self-assessed their levels of psychological and environmental empowerment. In other words, the study has involved a complete two-way interaction in employees’ psychological empowerment and environmental empowerment, examined in relation to in-role performance assessed by managers.

### 2.4 Sampling frame

Due to the unavailability of some units of analysis and the lack of information about the size and effect of sampling error, the study has utilized a non-probability sampling approach. Non-probability sampling has been an effective method to generate ideas and obtain feedback from readily available individuals or specific target groups. Cavana et al. (2001) indicate that purposive sampling entails selecting subjects who have been in the best position to provide the necessary information. Due to this study using match-pair design, we utilized match-pair purposive sampling to select subjects who are in the best position to provide the required information.

The judgment sampling has included managers and engineers who have been selected based on matched-pair relationships and direct interactions. The specific criteria for the judgment sampling of engineers have required them to hold a minimum diploma qualification to respond to the questionnaire and to have worked directly under the supervision of a specific manager for a minimum of 1 year. For the judgment sampling of managers, the criteria have required them to have worked with a particular engineer for a minimum of 1 year.

### 2.5 Sample size

G*Power is a program that can assist in conducting a power analysis and selecting a minimum sample size. According to Faul et al. (2007), G*Power can conduct distinct types of statistical tests, including *F*-tests, *t*-tests, *X*^2^-tests, and *z*-tests. In addition, G*Power is a statistical power analysis tool for multiple regression models (Cohen, 1992). The measurement model had to meet an acceptable threshold in terms of an indicator loading above the value of 0.70.

G*Power analysis was run to determine the sample size based on the research framework. The sample size (N) was calculated as a function of the required power level (1-*β*), the prespecified significance level α, and the population effect size to be detected with probability (1-*β*). *A priori* analysis provides an efficient program for controlling statistical power before a study is conducted (Bredenkamp and Erdfelder, 1985). To calculate the minimum sample size, the study employed a medium effect size of 0.15, α error probability of 0.05, and a minimum power of 0.80.

The results indicated a minimum sample size of 98 respondents to be considered large enough to run data analysis. In addition, there were two individual groups involved in the study, namely, managers and engineers. Therefore, the study had to collect a minimum sample of 98 managers and 98 engineers to ensure adequate statistical power.

### 2.6 Research instrument

There were two categories of respondents based on a matched-pair design; these were managers and engineers. To fulfil the requirements of a standard matched-pair approach, different instruments were designed for each group. Set A was developed for managers to assess engineers regarding in-role performance, and Set B was designed for engineers to respond to questions about psychological empowerment and environmental empowerment in the workplace.

Psychological empowerment instrument of Spreitzer (1995) was used to investigate perceived meaning, perceived competence, and perceived impact. Each of these dimensions measures three items, resulting in a total of nine items. For example, perceived meaning (PM) was measured as PM1, PM2, and PM3; perceived competence (PC) was measured as PC1, PC2, and PC3; and perceived impact (PI) was measured as PI1, PI2, and PI3. (Refer to Table 1 for item labels.) The nine-item instrument measured engineers’ psychological empowerment and was scored using a seven-point Likert scale, ranging from 1 (strongly disagree) to 7 (strongly agree).

**Table 1:** Measurement model.

Construct	Items	Loadings	AVE	CR
Set A: managers				
In-role performance	IRP		0.684	0.938
Engineer adequately performs assigned duties.	IRP1	0.851		
Engineer fulfils responsibilities specified in the job description.	IRP2	0.781		
Engineer performs tasks that are expected of him/her.	IRP3	0.873		
Engineer meets formal performance requirements of the job.	IRP4	0.807		
Engineer engages in activities that will directly affect his/her performance evaluation.	IRP5	0.830		
Engineer never neglects aspects of the job that he/she is obligated to perform.	IRP6	0.807		
Engineer never fails to perform essential duties.	IRP7	0.836		

Set B: engineers				
Perceived meaning	PM		0.874	0.954
The work I do is very important to me.	PM1	0.933		
My job activities are personally meaningful to me.	PM2	0.940		
The work I do is meaningful to me.	PM3	0.933		
Perceived competence	PC		0.856	0.947
I am confident about my ability to do my job.	PC1	0.910		
I am self-assured about my capabilities to perform my work activities.	PC2	0.945		
I have mastered the skills necessary for my job.	PC3	0.919		
Perceived impact	PI		0.876	0.955
My impact on what happens in my department is large.	PI1	0.920		
I have a great deal of control over what happens in my department.	PI2	0.961		
I have significant influence over what happens in my department.	PI3	0.927		
Work methods	WM		0.795	0.921
I am allowed to decide how to go about getting my job done (the methods to use).	WM1	0.987		
I am able to choose the way to go about my job (the procedures to utilize).	WM2	0.873		
I am free to choose the method(s) to use in carrying out my work.	WM3	0.905		
Work schedules	WS		0.833	0.937
I have control over the scheduling of my work.	WS1	0.900		
I have some control over the sequencing of my work activities (when I do what).	WS2	0.912		
My job is such that I can decide when to do particular work activities.	WS3	0.926		
Work criteria	WC		0.867	0.952
My job allows me to modify the normal we are evaluated so that I can emphasize some aspects of my job and play down others.	WC1	0.906		
I am able to modify what my job objectives are (what I am supposed to accomplish).	WC2	0.958		
I have some control over what I am supposed to accomplish (what my manager sees as my job objectives).	WC3	0.906		

CR, composite reliability; AVE, average variance extracted.

Environmental empowerment instrument used was based on Breaugh (1985) scale, originally designed to assess workers’ autonomous behavior. Meyerson (2007) and Meyerson and Kline (2008) adapted the Breaugh (1985) scale to assess environmental empowerment. The environmental empowerment scale measures three dimensions: work methods, work schedules, and work criteria. Each work dimension measured three items, to give a total of nine items. For example, work methods (WM) were measured using WM1, WM2, and WM3; work schedules (WS) were measured using WS1, WS2, and WS3; and work criteria (WC) were measured using WC1, WC2, and WC3. The instrument consisting of nine items was employed to measure engineers’ environmental empowerment and was scored using a seven-point Likert scale, ranging from 1 (strongly disagree) to 7 (strongly agree).

In-role performance (IRP) instrument used was adapted from Williams and Anderson, 1991. Managers utilized this instrument to assess engineers’ in-role performance due to the matched-pair study design. The instrument consists of seven items, labelled IRP1, IRP2, IRP3, IRP4, IRP5, IRP6, and IRP7. Engineers were requested to respond using a five-point Likert scale, ranging from 1 (never) to 5 (very often). Note that Items 6 and 7 are negatively worded and were revised to be positively worded items. Table 1 summarizes in-role performance items.

### 2.7 Data collection procedure

The data collection process was specifically designed to meet the requirements of the matched-pair design. We collected data from managers and engineers in Malaysian E&E manufacturing firms by utilizing a ‘walk-in, drop-off, and collect’ method and Pos Laju services. Manufacturing firms located in the Borneo states of Sabah and Sarawak received the instruments through Pos Laju services.

We contacted the human resource (HR) managers of the manufacturing firms to explain the study’s purpose and request cooperation in identifying relevant managers and engineers. The package included an official cover letter and 10 copies of the Set A questionnaires (each in a different color: red, orange, yellow, green, blue, purple, brown, white, crimson, and pink), along with 10 envelopes. Additionally, we attached 10 copies of the Set B questionnaires (each in a different color: red, orange, yellow, green, blue, purple, brown, white, crimson, and pink) to 10 envelopes, along with one self-addressed Pos Laju prepaid envelope. The cover letter emphasized that managers and engineers must be full-time workers who have worked together in a manufacturing firm for at least 1 year.

To enhance the response rate, we sent friendly reminders via letters, emails, and phone calls approximately 2 weeks after initially distributing the questionnaires. Out of the 274 firms approached, a total of 73 agreed to participate after engaging in productive discussions with HR managers.

To mitigate workplace favoritism biases effectively, we engaged human resource managers in the data collection process, entrusting them with the task of methodically pairing managers and engineers. This approach ensured a balanced representation of perspectives. For a coherent and organized collection, we distributed questionnaires employing a unique color-coding system, assigning each manager-engineer pair a distinct color for easy identification and consistency. Following this systematic approach, human resource managers efficiently collected responses, adhering to the designated color scheme, thus maintaining the integrity and orderliness of the data gathering process.

### 2.8 Statistical analysis: SPSS statistics, SPSS Amos, and SmartPLS

We have employed the Statistical Package for the Social Sciences (SPSS) Statistics, SPSS Amos, and SmartPLS to conduct analyses. To gain an overview of the data, we have used SPSS Statistics to compute frequency, percentage, mean score (M), standard deviation (SD), and the expectation–maximization algorithm (EM).

For analyzing the model fit of the individual cognitive empowerment construct, we have utilized SPSS Amos to conduct confirmatory factor analysis (CFA). The assessment has included several fit indices, namely the minimum fit function χ^2^ (CMIN chi-square), degrees of freedom (*df*), comparative fit index (CFI), root mean square error of approximation (RMSEA), and standardized root mean square residual (SRMR).

Additionally, we have utilized SmartPLS for structural model analysis to test composite reliability (CR), indicator loading, average variance extracted (AVE), heterotrait-monotrait ratio of correlations (HTMT), variance inflation factor (VIF), tolerance, effect size (*f*^2^), coefficient of determination (*R*^2^), and the direct effect framework between environmental empowerment and in-role performance, including the beta value (*β*), standard error, and *t-value*.

## 3 Results

### 3.1 Respondent profile

A total of 173 pairs of questionnaires have been collected from E&E manufacturing firms in Malaysia. Missing data has been handled using the EM algorithm. Respondents who failed to answer more than 15 percent of the questionnaire items have been excluded from the analysis (Hair et al., 2017). Missing data below 15 percent of the total questions was not present in the study. All 173 pairs have been deemed usable for data analysis. The sample has comprised matched pairs of 173 managers (Set A) and 173 engineers (Set B) from five states in Malaysia: Johor, Selangor, Perak, Penang, and Sabah.

### 3.2 Mean score and standard deviation

The study has revealed a positive response to the items in Set A, as evidenced by a mean score (M) of 4.174 and a low standard deviation (SD) of 0.772, indicating a high level of agreement among participants.

In Set B, the M and SD have suggested a range of perceptions regarding the four dimensions assessed: perceived meaning, competence, impact, and work methods. For perceived meaning (*M* = 5.567, SD = 1.123), competence (*M* = 5.543, SD = 1.093), and impact (*M* = 5.347, SD = 1.191), the mean scores have been above the midpoint of the scale, suggesting moderate to high levels of agreement. However, for work methods (*M* = 5.245, SD = 1.126), the mean score has been closer to the midpoint, indicating a more neutral perception among participants.

Regarding work schedules (*M* = 4.811, SD = 1.466) and criteria (*M* = 4.879, SD = 1.113), the mean scores have fallen below the midpoint of the scale, suggesting a slight disagreement among participants. These findings indicate that perceptions of work schedules and criteria have been less favorable than those of the other dimensions.

### 3.3 Common method bias

We have conducted a lateral collinearity test to assess the presence of multicollinearity among the predictor variables. Referring to Table 2, we have examined the variance inflation factor (VIF) and tolerance values for each predictor variable. The VIF scores have ranged from 2.270 to 3.239, all falling below the commonly accepted threshold of 3.3, which indicates severe multicollinearity (Kock, 2015). Additionally, the tolerance values have ranged from 0.327 to 0.442, exceeding the recommended minimum value of 0.20 (Menard, 1995).

**Table 2:** Result of direct effect.

Relationship	VIF tolerance	*β*	Std error	*t*-values	*p*-values	*f* ^2^	*R^2^*	Decision
[H2] WM → IRP	3.239 0.312	0.300	0.081	3.721***	0.000	0.095	0.708	Supported
[H3] WS → IRP	2.270 0.442	−0.037	0.058	0.642	0.261	0.002	0.708	Not supported
[H4] WC → IRP	3.083 0.327	0.064	0.071	0.896	0.185	0.004	0.708	Not supported

*n* = 173; VIF < 5.0; tolerance > 0.2; ***p < 0.01; **p < 0.05; *p < 0.10; 5,000 resampling bootstrapping; one-tailed procedure.

### 3.4 Composite reliability

Composite reliability (CR) has assessed the internal consistency reliability of the constructs, with a recommended threshold of 0.70 or higher (Gefen et al., 2000). The CR values for all constructs have surpassed the recommended threshold of 0.70, indicating that the measurement scales exhibit satisfactory internal consistency. Table 1 summarizes these results.

### 3.5 Convergent validity

The indicator loading values for all constructs have exceeded 0.708, indicating effective measurement of the underlying construct. The AVE values for all constructs have surpassed 0.50, demonstrating consistent measurement of the construct. The CR values for all constructs have exceeded 0.70, confirming the reliability of the constructs. These results suggest the validity and reliability of the measurement model (Hair et al., 2017).

### 3.6 Discriminant validity

The study has assessed discriminant validity using the heterotrait-monotrait ratio of correlations (HTMT). The HTMT criterion has served as a marker for establishing discriminant validity. An HTMT value below 0.90 has established discriminant validity between two reflective constructs (West et al., 2012). The study has constructed a confidence interval for the HTMT, which has shown none of the intervals below the value of 0.90, thereby indicating no discriminant validity issues. Refer to Table 3.

**Table 3:** Heterotrait-monotrait ratio of correlations (HTMT).

Variables	1	2	3	4	5	6	7
1. Work criteria	**1.000**						
2. Work methods	0.810 CI 0.90 (0.438, 0.763)	**1.000**					
3. Work schedules	0.777 CI 0.90 (0.528, 0.805)	0.763 CI 0.90 (0.683, 0.877)	**1.000**				
4. In-role performance	0.719 CI 0.90 (0.419, 0.744)	0.835 CI 0.90 (0.757, 0.905)	0.599 CI 0.90 (0.683, 0.0.889)	**1.000**			
5. Perceived competence	0.753 CI 0.90 (0.370, 0.693)	0.771 CI 0.90 (0.582, 0.820)	0.601 CI 0.90 (0.583, 0.811)	0.826 CI 0.90 (0.513, 0.775)	**1.000**		
6. Perceived impact	0.728 CI 0.90 (0.424, 0.749)	0.781 CI 0.90 (0.648, 0.856)	0.609 CI 0.90 (0.734, 0.907)	0.813 CI 0.90 (0.675, 0.881)	0.820 CI 0.90 (0.719, 0.869)	**1.000**	
7. Perceived meaning	0.679 CI 0.90 (0.319, 0.619)	0.808 CI 0.90 (0.464, 0.723)	0.598 CI 0.90 (0.445, 0.713)	0.806 CI 0.90 (0.463, 0.710)	0.844 CI 0.90 (0.678, 0.853)	0.848 CI 0.90 (0.652, 0.827)	**1.000**

Confidence interval bias corrected is shown (5.0, 95%); CI, confidence interval; HTMT discriminates at < 0.9.

### 3.7 Structural model assessment: model fit of individual cognitive empowerment

The study has used confirmatory factor analysis (CFA) to evaluate invariance models, focusing on the instrument’s authenticated psychological factors and environmental factors through item-factor analysis. The CMIN/*df* value, indicating the discrepancy divided by the degrees of freedom, was significantly higher at 6.317 for the one-factor model (psychological empowerment without environmental empowerment) compared to the two-factor model (psychological empowerment with environmental empowerment), which had a notably lower CMIN/*df* value of 4.490. According to Marsh and Hocevar (1985), a CMIN/*df* value of ≤5 discovers a reasonable fit, thus endorsing the structure of the two-factor model (psychological empowerment with environmental empowerment).

The comparative fit index (CFI) has further confirmed the enhanced fit of the two-factor model (psychological empowerment with environmental empowerment), showing a value of 0.846. West et al. (2012) recommend, values closer to 1.000 indicate a better fit. Additionally, the normed fit index (NFI), which ranges between 0 and 1, has been higher for the two-factor model (psychological empowerment with environmental empowerment).

Values of RMSEA and SRMR closer to 0 suggest a better fit. For RMSEA, a value of 0.05 or less generally indicates a good fit, while a value of 0.10 or less suggests a reasonable fit. For SRMR, a value of 0.08 or less generally indicates a good fit, while a value of 0.10 or less suggests a reasonable fit. The one-factor model’s RMSEA and SRMR values have both exceeded 0.1, indicating a poor fit. Meanwhile, the two-factor model has demonstrated lower RMSEA and SRMR values, indicating a better fit. Therefore, Hypothesis H1 is supported, and the two-factor model (psychological empowerment with environmental empowerment) is considered a more optimal fit for measuring individual cognitive empowerment, as shown in Table 4 below.

**Table 4:** Model fit measures of empowerment fit indicating the one-factor model and two-factor model.

**Fit index**	**One-factor model**	**Two-factor model**
Minimum fit function *X^2^*	852.841 (135), *p* < 0.000	601.71 (134), *p* < 0.000
CMIN/*df*	6.317	4.490
Comparative fit index (CFI)	0.764	0.846
Root mean square error of approximation (RMSEA)	0.176	0.142
Standardized root mean square residual (SRMR)	0.084	0.063

CMIN, chi-square; df, degrees of freedom.

Structural model assessment: environmental empowerment and in-role performance The study has employed a bootstrapping procedure with 5,000 resamples, Wong (2013) recommended, to assess the one-tailed direct effect and determine the statistical significance of the relationships between environmental empowerment factors, particularly WM, WS, and WC on IRP. We have conducted the bootstrapping procedure using SmartPLS software. We have utilized critical values for significance levels of 1, 5, and 10%, corresponding to *t-value*s for one-tailed tests of 2.330, 1.645, and 1.280 (Hair et al., 2017).

We have evaluated the effect size (*f*^2^) using cut-off values of 0.02, indicating a small magnitude effect, 0.15 for a moderate magnitude effect, and 0.35 for a strong magnitude effect (Cohen, 1988). The *f*^2^ values for WM→IRP, WS→IRP, and WC→IRP have been 0.095, 0.002, and 0.004, suggesting a small magnitude effect for WM and no magnitude effect for WS and WC.

The *R*^2^ value, representing the proportion of variance in IRP explained by the WM, WS, and WC, has been 0.708, indicating that 70.8% of the variance in IRP. According to Chin (1988), an *R*^2^ value of 0.67 or higher is considered substantial, a value between 0.33 and 0.66 is moderate, and a value below 0.33 is weak. The *R*^2^ value obtained in the study suggests that the model provides a substantial explanation for IRP. Please refer to Table 2 for detailed results.

The bootstrapping procedure has revealed a positive and significant direct effect of WM on IRP (*β* = 0.300, ***p < 0.01), providing support for Hypothesis H2. The finding indicates that WM have a statistically significant and positive relationship on IRP, suggesting that employees who perceive greater autonomy and control over their WM tend to exhibit higher *p*-value significant levels of IRP.

However, the relationships between WS (*β* = −0.037, *p* > 0.10) and WC (*β* = 0.064, *p* > 0.10) and IRP were not found to be statistically significant, suggesting that these two environmental empowerment factors did not have a significant influence on IRP. Therefore, Hypotheses H3 and H4 were not supported.

## 4 Discussion

### 4.1 Mean scores and standard deviation (SD): research instruments

The study has investigated two sets of instruments designed to assess constructs related to work-related well-being. Set A has comprised items measuring perceived meaning, perceived competence, and perceived impact, while Set B has consisted of items assessing work methods, work criteria, and work schedules. The analysis of participants’ responses has determined mean scores (M) and standard deviations (SD).

The M for Set A’s items have all been positive, ranging from 4.62 to 5.41, indicating that participants have generally found the items relevant and well-written. Additionally, the SD for Set A’s items have been relatively low, ranging from 0.78 to 1.12, suggesting a high level of agreement among participants.

The M for the six variables in Set B have ranged from 4.811 to 5.567, reflecting a generally positive response. However, the SD for these variables have ranged from 1.093 to 1.466, indicating some inconsistency in responses. Notably, perceived meaning and perceived competence have shown the highest M (5.567 and 5.545), while work criteria and work schedules have had the lowest M (4.811 and 4.922).

The findings suggest that the instruments are well-suited for assessing the targeted constructs. The positive M and low SD in Set A indicate that participants have found these items relevant and well-written, with a high level of agreement in responses. The positive M in Set B suggests a generally favorable perception of the assessed work methods, work criteria, and work schedules. However, the variability in responses, indicated by the higher SD in Set B, suggests that further research is necessary to understand the factors contributing to this variability.

### 4.2 Re-examining the two-factor model fit of individual cognitive empowerment: psychological and environmental factors

The study has supported the hypothesis that the two-factor model of individual cognitive empowerment, encompassing psychological and environmental empowerment, provides an optimal fit, unlike the one-factor model, which only includes psychological empowerment. The CFA results have validated a significant fit for the two-factor model. This conclusion holds significant implications for measuring individual cognitive empowerment and emphasizes the need to integrate both psychological and environmental empowerment considerations.

Meyerson (2007) and Meyerson and Kline (2008) have described two fundamental components in relation to the model fit of individual cognitive empowerment: intrinsic motivational factors (psychological empowerment) and relational factors (environmental empowerment). Intrinsic motivational factors are linked to an individual’s psychological empowerment, reflecting internal feelings of individual cognitive empowerment. On the other hand, relational factors relate to an individual’s environmental empowerment, indicating the influence sphere within workplace conditions, including power delegation or transitions. The study has reshaped the two-factor model fit of individual cognitive empowerment by integrating three dimensions of psychological empowerment (perceived meaning, perceived competence, and perceived impact) and three aspects of environmental empowerment (work methods, work criteria, and work schedules), while displacing the self-determination component of psychological empowerment.

Furthermore, the study has conducted a matched-pair examination to reassess the model fit of the individual cognitive empowerment instrument, corroborating the model’s efficacy in assessing individual cognitive empowerment. The two constructs did not specifically measure individual cognitive empowerment. Breaugh’s measurement scale (1985), relating to environmental working conditions, has been assessed and deemed an apt approach to evaluate empowering environmental factors, replacing self-determination with psychological empowerment.

### 4.3 Relationship between environmental empowerment and in-role performance

The study has aimed to investigate the influence of work methods, work schedules, and work criteria on the in-role performance of engineers. The results have highlighted a substantial positive regression between the work methods engineers utilize and their ability to perform professional duties effectively. Notably, we have understood a positive relationship between social exchange interactions involving engineers and managers and in-role performance from the managers’ viewpoint. This has emphasized the importance of effective adoption and understanding of work methods between managerial personnel and engineers in achieving superior job outcomes.

The study has not substantiated the hypothesis that work schedules and work criteria have a considerable impact on in-role performance. This departure from prior research by Al-Mekhlafi et al. (2022), Dong et al. (2022), Kandie and Chepkilot (2022), Lau and Aguirre Reid (2022), and Misnan et al. (2022) suggests that complying with a fixed schedule and meeting certain criteria can augment job performance and requires further study. A plausible reason for this discrepancy might be the inherent variability in engineers’ compliance with work schedules and work criteria, compared to other professionals. This variability might originate from the potentially unpredictable or autonomous nature of engineering work. From a management perspective within the engineering industry, the focus often lies on operational effectiveness rather than the effects of work schedules and work criteria on in-role performance.

The findings have emphasized the necessity to comprehend the distinctive factors affecting in-role performance among engineers, which can then inform managerial strategies to enhance employee productivity. Consequently, the study has broadened in understanding of the factors influencing in-role performance within the engineering sector. To understand the intricate relationship between work methods, social exchange between managers and engineers, and in-role performance, the study has paved the way for strategies aimed at optimizing productivity.

Other unique factors that have affected in-role performance among engineers are the specific nature of the work and the particular work methods utilized.

### 4.4 Theoretical contributions and implications

The study has made a theoretical contribution by incorporating a two-factor model of individual cognitive empowerment and combining frameworks of psychological empowerment and environmental empowerment. To integrate these two dimensions, the study has provided a more comprehensive understanding of internal psychological factors and external environmental factors that interact to influence an individual’s cognitive development. This theoretical framework has enhanced the existing literature on environmental empowerment and provided a holistic perspective for examining the impact of individual cognitive empowerment on in-role performance.

### 4.5 Practical contribution and implication

The findings of the study have highlighted the importance of the work environment and autonomy for engineers through their work methods. This autonomy of work methods has appeared to significantly enhance in-role performance, suggesting that the engineers’ ability to select methods, procedures, and strategies has regressed positively with in-role performance. Organizations should consider revising their management practices to allow engineers more control over how engineers approach tasks. Empowering engineers have a sense of ownership and trust, nurturing an environment conducive to innovation and continuous improvement. Organizations can build a more committed and motivated employee through decentralizing decision-making processes and demonstrating trust in their employees’ expertise. This can lead to higher job performance, satisfaction, then reduced turnover rates. Such autonomy has harnessed engineers’ skill sets and knowledge more effectively, thereby yielding beneficial outcomes for both the individuals and the organization. To harness this potential, organizations should create a culture that encourages experimentation and rewards innovative thinking. Organizations should provide opportunities for engineers to continuously update and expand their skill sets. This can be achieved through targeted training programs, access to the latest tools and technologies.

The study has revealed a negative relationship between engineers’ work schedules and their in-role performance. Despite this, manufacturing firms have the opportunity to reduce this negative impact strategically implementing flexible work arrangements and comprehensive time-off policies (Van Cutsem et al., 2017; Pérez-Pérez et al., 2019). To provide engineers with more control over their work schedules while adhering to company policies, firms can empower employees to cultivate a sense of ownership and control over their tasks (Beutell, 2010). It is important to note that engineers often work irregular hours, including on-call shifts, which can impact their alertness and job dedication (Pilcher et al., 2002). Additionally, access to flexible schedule options may be limited for less privileged workers (Swanberg et al., 2005). Companies must consider the needs and preferences of their engineers when designing work schedules (Bergman et al., 2023). In acknowledging the importance of balancing flexibility with explicit boundaries, manufacturing firms can enhance operational efficiency and achieve superior outcomes.

In response to the perceived worthlessness of engineers’ work criteria on job performance, the management has taken actionable steps. These include setting up transparent communication channels between managers and engineers, conducting regular performance assessments, involving engineers in collective goal-setting processes, providing training and development opportunities, promoting a culture of continuous improvement, and re-evaluating the evaluation methodology. The software engineering community has widely accepted these practices and measures, linking them to knowledge activities (KAs) for implementing knowledge management in software development teams (Dietze and Kahrens, 2024). Additionally, individual continuous learning has positively impacted engineers’ risk tolerance and self-efficacy (Liu et al., 2020). To adopt these strategies, engineers have developed a comprehensive understanding of expectations, affiliated innovations with organizational objectives, improved their skills, and made more significant contributions, thereby elevating the perceived importance of their work within job descriptions and achieving superior outcomes.

Creating a work environment that supports flexible work arrangements has improved managers’ and engineers’ work-life balance (Dizaho et al., 2017; Kabir et al., 2023), has reduced absenteeism rates, and has facilitated their maintenance of good health (Shifrin and Michel, 2022). However, as flexible work arrangements have given employees more control over their work schedules, implementing this policy cautiously is essential because it has the potential to reduce employee engagement (vigor, dedication, absorption) (Timms et al., 2015). Therefore, setting up a measurable performance management system and conducting rigorous evaluations have become crucial. These efforts require high commitment from both employees and organizations and substantial organizational support, as flexible work arrangements have emerged as one of the best methods to attract and retain talent (Smith et al., 2019).

The study has revealed that engineers have prioritized creating individual cognitive empowerment, focusing on enhancing work schedules and work criteria that have adversely impacted in-role performance. Given the deterioration in work schedules and work criteria for engineers, it is recommended that management actively devise solutions to improve work schedules and establish a broad set of criteria for practices. Encouraging flexibility in these domains has counterbalanced the negative effects of insufficient work principles on organizational performance. This could involve adopting flexible work arrangements or guidelines that prioritize engineers’ performance outcomes.

### 4.6 Limitations and directions for future research

The study has encountered certain limitations, primarily due to its limited sample size of 173 and the exclusive inclusion of 73 E&E manufacturing firms. This aspect has potentially curtailed the generalizability of the findings. To amplify the representativeness of the results, subsequent investigations should consider enlarging the sample size and incorporating a broader spectrum of firms across diverse industries. Furthermore, to augment the extrapolation of findings across other contexts, we should contemplate examining the results in varied industrial sectors and countries. Such an attempt would unveil potential disparities in work methods, work schedules, and work criteria across distinct contexts, subsequently offering perceptions into the impact on in-role performance. In essence, while the study has offered meaningful perceptions into the interplay between work methods, work schedules, and work criteria on in-role performance within the E&E manufacturing sector, it necessitates the replication and expansion of these findings in alternative contexts in future research.

Another limitation has lain in its singular focus on individual cognitive empowerment, encompassing the totality of empowerment experiences that engineers have undergone. Social or structural empowerment might play a pivotal role in shaping a sense of workplace empowerment. Future studies should consider examining other empowerment dimensions or factors to garner a more holistic understanding of the concept. Concurrently, we might employ additional measures or techniques to mitigate biases in the labeled data (Liu et al., 2020) and discrimination in many areas of social life can be effects potentially influencing the outcomes (Lewis, 2023). For instance, the utilization of multiple data sources, surveys, interviews, and observations could aid in triangulating and validating the findings, thereby ensuring a more precise portrayal of engineers’ experiences relating to empowerment. To summarize, while the study holds significant implications for the E&E industry by emphasizing the role of cognitive empowerment in fostering in-role performance among its professionals, further research is warranted to delve deeper into the concept of individual cognitive empowerment. This examining could consider other empowerment dimensions or factors and employ additional measures or techniques to curb biases and social desirability effects.

The findings of the study have suggested that the two-factor empowerment model has outperformed the one-factor model. However, the fit indices for the two-factor model have still been below acceptable standards, signifying that the present model has not entirely accounted for all factors contributing to the empowerment concept. Future research could refine the two-factor model or probe additional factors influencing empowerment to enable a more comprehensive understanding of the concept. To address this limitation, we might examine other potential contributors to the empowerment concept, namely, social and political empowerment factors (Aleshinloye et al., 2022). To refine the two-factor model to encompass other empowerment dimensions or to adjust the factors to better reflect the experiences of E&E professionals, a more comprehensive understanding of empowerment and its effects on in-role performance could be developed. This understanding could shape organizational policies and practices aimed at promoting employee empowerment and in-role performance within the E&E industry. Thus, addressing this limitation holds significant potential for the field and could have practical implications for E&E manufacturing firms.

Despite the appropriate use of Breaugh’s measurement scale (1985) for assessing environmental work conditions, it may not have fully captured the intricacies of environmental empowerment. To provide a more understanding of this concept, future studies should probe additional factors or dimensions of environmental empowerment, including organizational culture, organizational management systems, transformational leadership, and organizational identification (Shahbazian and Beheshtifar, 2020; Bose et al., 2021). Investigating these factors or dimensions could identify other contributors to engineers’ sense of empowerment within their work environment. Past studies have found that organizational culture, organizational management systems, transformational leadership, and organizational identification significantly influence the promotion or inhibition of environmental empowerment. For instance, green transformational leaders who empower their subordinates have led to employees’ discretionary behavior towards the environmental management of the organization, denoted as organizational environmental citizenship behavior (Priyadarshini et al., 2023). Additionally, psychological empowerment has mediated the relationship between transformational leadership and employee outcomes, such as organizational citizenship behavior and turnover intention (Saira et al., 2020; Bose et al., 2021). Therefore, it is important for organizations to consider these factors in promoting environmental empowerment among employees.

## 5 Conclusion

The study has provided perceptions into the central tendency and variability of datasets, indicating that participants have generally responded positively to the scale items, though with some variability in responses for each variable. It is necessary to emphasize the importance of considering multiple factors such as perceived meaning, competence, impact, work methods, criteria, and schedules in evaluating participants’ responses.

The study has highlighted the significance of the work environment and autonomy for engineers and their work methods, which have appeared to significantly enhance in-role performance and effectiveness. However, the study has revealed a negative regression between engineers’ work schedules and in-role performance. The study suggests that engineers should prioritize creating individual cognitive empowerment, focusing on enhancing work schedules and criteria that negatively affect in-role performance.

The findings of the study have revealed that the two-factor empowerment model has improved compared to the one-factor model. However, the model fit indices for the two-factor model are still below acceptable standards, signifying that the current model does not fully justify all factors contributing to the empowerment concept. Future research could refine the two-factor model or investigate additional factors influencing empowerment to enable a more comprehensive understanding of the concept.

## Data Availability

The raw data supporting the conclusions of this article will be made available by the authors, without undue reservation.
